# Generation of Premature Termination Codon (PTC)-Harboring Pseudorabies Virus (PRV) via Genetic Code Expansion Technology

**DOI:** 10.3390/v14030572

**Published:** 2022-03-10

**Authors:** Tong-Yun Wang, Guo-Ju Sang, Qian Wang, Chao-Liang Leng, Zhi-Jun Tian, Jin-Mei Peng, Shu-Jie Wang, Ming-Xia Sun, Fan-Dan Meng, Hao Zheng, Xue-Hui Cai, Yan-Dong Tang

**Affiliations:** 1 State Key Laboratory of Veterinary Biotechnology, Harbin Veterinary Research Institute, The Chinese Academy of Agricultural Sciences, Harbin 150069, China; sdndwty@163.com (T.-Y.W.); wangqian@caas.cn (Q.W.); tianzhijun@caas.cn (Z.-J.T.); pengjinmei@caas.cn (J.-M.P.); wangshujie@caas.cn (S.-J.W.); sunmingxia@caas.cn (M.-X.S.); mengfandan@caas.cn (F.-D.M.); 2 Shanghai Veterinary Research Institute, The Chinese Academy of Agricultural Sciences, Shanghai 200241, China; 18438615167@139.com; 3 Henan Provincial Engineering and Technology Center of Animal Disease Diagnosis and Integrated Control, Nanyang Normal University, Nanyang 473061, China; lenghan1223@126.com

**Keywords:** premature termination codon, pseudorabies virus, genetic code expansion

## Abstract

Despite many efforts and diverse approaches, developing an effective herpesvirus vaccine remains a great challenge. Traditional inactivated and live-attenuated vaccines always raise efficacy or safety concerns. This study used Pseudorabies virus (PRV), a swine herpes virus, as a model. We attempted to develop a live but replication-incompetent PRV by genetic code expansion (GCE) technology. Premature termination codon (PTC) harboring PRV was successfully rescued in the presence of orthogonal system MbpylRS/tRNA^Pyl^ pair and unnatural amino acids (UAA). However, UAA incorporating efficacy seemed extremely low in our engineered PRV PTC virus. Furthermore, we failed to establish a stable transgenic cell line containing orthogonal translation machinery for PTC virus replication, and we demonstrated that orthogonal tRNA^Pyl^ is a key limiting factor. This study is the first to demonstrate that orthogonal translation system-mediated amber codon suppression strategy could precisely control PRV-PTC engineered virus replication. To our knowledge, this is the first reported PTC herpesvirus generated by GCE technology. Our work provides a proof-of-concept for generating UAAs-controlled PRV-PTC virus, which can be used as a safe and effective vaccine.

## 1. Introduction

Developing the herpesviruses vaccine is challenging because of the immunologically silent nature of its latency, and the virus mediates immune evasion [1,2]. Pseudorabies virus (PRV) is a swine herpesvirus, also known as Aujeszky virus, which belongs to the genus *Varicellovirus* in the subfamily *Alphaherpesvirinae* of the family *Herpesviridae* [3]. PRV is lethal to many domestic and wild animals, and pigs are the natural host [4]. Since 2011, PRV variants have emerged in China, and commercial vaccines fail to provide complete protection against PRV [5,6]. More seriously, PRV variants can spill over into humans and cause severely nerve-related diseases [7,8]. Developing a safe and effective PRV vaccine is one of the best choices for PRV control in related animals and humans.

Inactivated vaccines play a vital role in eradicating PRV in swine farms. However, the inactivated PRV vaccine mainly induces a humoral immune response, lacks effective T cell response, and inactivated PRV vaccines fail to stop viral shedding post-virus challenge [9,10]. Live-attenuated vaccines have shown the best efficacy against PRV; however, this raises safety concerns, e.g., the attenuated PRV strains are lethal to dogs and can spread horizontally [11]. Therefore, developing a safe and effective PRV vaccine faces a dilemma.

**Figure 1:**
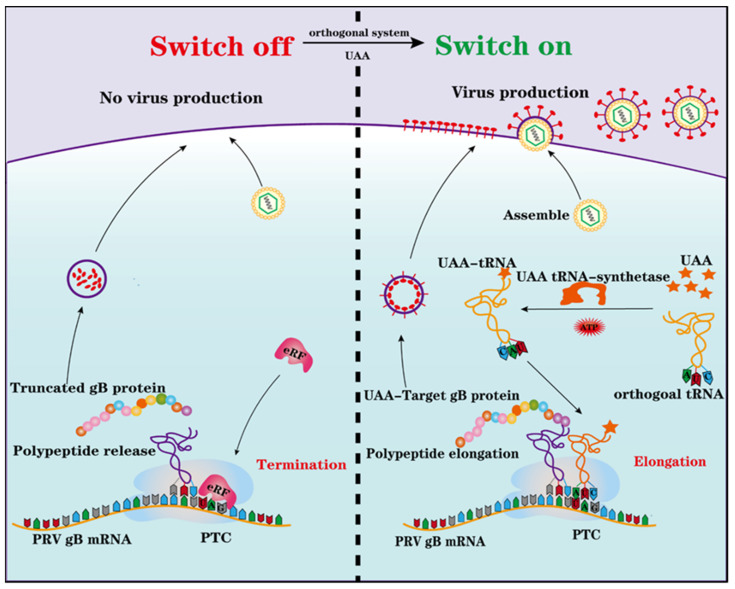
Schematic representation of the construction of PTC harboring PRV via genetic code expansion. PTC harboring PRV failed to replicate in normal cells (**Left**) and can replicate in cells with orthogonal translation machinery system (**Right**). UAA, unnatural amino acid. PTC, premature termination codon.

Genetic code expansion (GCE) technology is an orthogonal translation system derived from the *Methanosarcina barkeri*. In this microbe, amber (TAG) stop codon can be read-through with the cooperation of Mb pyrrolysyl tRNA synthetase/tRNA^Pyl^ pair (MbpylRS/tRNA^Pyl^) and unnatural amino acids (UAA) [12,13,14]. The application of GCE technology in PTC harboring PRV is illustrated in Figure 1. The GCE technology provides a novel strategy to generate a live but replication-defective candidate vaccine. This technology has been successfully applied in the influenza A virus vaccine [14].

This study used PRV as a herpesvirus model and attempted to engineer a PTC site in an essential gene of PRV, *gB*, with amber codons (TAG). PTC harboring PRV could be successfully rescued in the orthogonal translation machinery system MbpylRS/tRNA^Pyl^ pair and UAA. However, UAA incorporating efficacy seemed extremely low in our engineered PRV PTC virus. Furthermore, all our attempts to construct cell lines containing orthogonal translation machinery system MbpylRS/tRNA^Pyl^ pair failed. These results suggested that several key issues should be resolved in PTC harboring herpesvirus production in the future.

## 2. Materials and Methods

### 2.1. Cells and Plasmids

Human embryonic kidney 293T cells (HEK-293T, ATCC CRL-11268), Rabbit kidney cells (RK13, ATCC CCL-37), Vero cells (ATCC CCL-81), Human embryonic kidney cells (HEK-293A, ATCC CRL-1573), swine testicular cells (ST, ATCC CRL1746), and Porcine kidney cells (PK15, ATCC CCL-33) were maintained in DMEM (Gibco, Waltham, MA, USA) with 10% (*v*/*v*) fetal bovine serum (ExCell Bio, Canberra, Australia). As previously described, the pseudorabies virus infectious clone was pBac-JS2012 [15]. Plasmids related to orthogonal translation system, pSD31-pylRS, bjmu-12t-zeo, and pSD31-GFP^39TAG^ were kindly provided by Professor De-Min Zhou of Peking University [14]. PiggyBac transposon plasmids pB513B and PB220A plasmids were described in our previous works [16,17].

### 2.2. Construction of PTC Harboring gB Mutants

pCAGGS gB plasmid was previously described [18]. The amino acids at positions 149Q, 169K, 171K, 177K, 185W, 206Q, 217K, 221K, 267K, 285W, 319H, 331Q, 362W, 365W, 367W, 370K, 379K, and 413Q of gB protein were mutated to amber codon TAG. Briefly, the indicated amino acid codon was replaced by a TAG amber codon by site-directed mutagenesis PCR. All clones were verified by DNA sequencing. The site-directed mutagenesis primers are listed in Supplementary Table S1.

### 2.3. Read-Through Efficacy for PTC Harboring gB by GCE

HEK-293T cells in good growth condition were plated in 24 well plates (4 × 10^5^ cells/well). 0.5 µg of MbpylRS/tRNA^Pyl^ plasmids were co-transfected with 0.5 µg of pCAGGS gB PTC plasmid using the jetPRIME transfection reagent (Polyplus, Illkirch, France). Two parallel experiments were conducted. pCAGGS gB was used as a positive control, and non-transfected cells were used as mock. The supernatant was replaced by fresh medium supplemented with 2% FBS in the presence of 1 mM NAEK (TRC, Ottawa, ON, Canada) or not, 6 h post-transfection. At 48 h post-transfection, a Western blot was performed to analyze the read-through efficacy of PTC harboring gB mutants. The cells were lysed by 70 µL lysis buffer (50 mM KCl, 100 mM NaCl, 0.25% NP-40, 1 mM DTT, and 50 mM herpes-NaOH) containing 1% protease inhibitor (Sigma-Aldrich, St. Louis, MO, USA) for 30 min on ice and then centrifuged at 12,000× *g* for 10 min at 4 °C. Cell lysates were mixed with 5× loading buffer and boiled at 100 °C for 10 min. As previously described, the samples were separated by 10% SDS-PAGE and transferred to polyvinylidene fluoride (PVDF) membranes [16].

### 2.4. Read-Through Efficacy for gB PTC Identified by Cell-to-Cell Fusion Assay

Cell-to-cell fusion assay was performed as previously described [18]. Briefly RK13 cells were placed in 24 well plates (4 × 10^5^ cells/well), and 0.15 μg pCAGGS gB or indicated gB PTCs, 0.15 μg pCAGGS gD, 0.15 μg pCAGGS gH, 0.15 μg pCAGGS gL, 0.15 μg pDC315-GFP, and 0.4 µg MbpylRS/tRNA^Pyl^ were co-transfected. The non-transfected cells were used as control. The supernatant medium was replaced by a fresh medium supplemented with 2% FBS in the presence of 1 mM UAA (NAEK) or not, 6 h post-transfection. 48 h after transfection, fluorescence microscopy was used to analyze the read-through efficacy by indicated gB mediated cell fusion analysis.

### 2.5. PTC Harboring PRV Construction

PRV-PTC construction was performed as previously described [15]. Briefly, the procedure is as follows. A DNA fragment with a galK expression cassette flanked by 50 bp homologous of *gB* gene was amplified by PCR using the primers gB-galKF/gB-galKR (gB-galKF: 5-GGGACCGCTTCTACGTCTGCCCGCCGCCGTCCGGCTCCACGGTGGT cctgttgacaattaatcatcggca-3; gB-galKR: 5-AGGCGGTCACCTTGTGGTTGTTGCGCACGTACTCGGCCTTGGAGACGCACTTGCCtcagcactgtcctgctcctt-3) and KOD DNA polymerase (Toyobo, Osaka, Japan). The obtained PCR product was digested with DpnI (Thermo Fisher, Waltham, MA, USA) at 37 °C for 1 h to remove the original template plasmid, followed by agarose gel purification. To generate SW102-JS-galK, 25 µL SW102-JS electrocompetent cells were prepared and electro-transformed with 100 ng galK DNA fragment under the condition of 1.5 kV, 25 μF, 200 Ω. Then, 800 µL SOC medium was added immediately after electro-transformation and incubation at 32 °C, 200 rpm for 1.5 h. The recovered bacteria were washed twice with 1 mL M9 solution and took 150 µL M9 solution to plate the bacteria cells onto M63 plates containing galactose and chloramphenicol. PCR was used to confirm galK positive colonies with primers LgalKup/LgalKdown (LgalKup: 5-TGCTGCGCCTCGACCCCAA-3; LgalKdown: 5-AAGAACTTAACCCGGCACCCT-3). The galK positive colonies were further screened on a MacConkey plate containing chloramphenicol to realize the nucleotide substitution of galK at positions 141 to 187 of gB in pBac-JS2012. Finally, gB fragments harboring PTC points were used to remove the *galK* gene from pBac-JS2012-galK. A DNA fragment with PTC points in gB ORF was amplified from the template gB mutant plasmids with the primers gB-LF/gB-RR (gB-LF: 5-cgacggtatcgataagcttgatCGCTGGTGGCGGTCTTTG-3; gB-RR: 5-ccgggctgcaggaattcgatGAGTCCAGGTCGATGGGGTAG-3). The obtained PCR product was also digested with DpnI (Thermo Fisher, Waltham, MA, USA) at 37 °C for 1 h. The indicated PTC harboring gB fragment was electro-transformed into SW102-JS-galK as described above. After 3 h at 32 °C, the transformed cells were washed and suspended in an M9 medium. The positive clone with mutant gB fragment replacing galK was screened on M63 minimal medium plates containing chloramphenicol, 2-deoxy-galactose, and glycerol. The obtained PRV Bac clone with TAG PTC in gB was termed PRV-PTC.

### 2.6. Rescue of PRV-PTC Virus

HEK-293T cells and Vero cells (2 × 10^6^) were plated in 6-well plates in DMEM supplemented with 10% FBS. Then, 2 µg MbpylRS/tRNA^Pyl^ plasmid were co-transfected with the 2 µg pPRV-Bac or the indicated pPRV-PTC-Bac using the transfection reagent jetPRIME (Polyplus, Illkirch, France) according to the manufacturer’s instructions. At 6 h post-transfection, the supernatant was replaced with DMEM containing 2% FBS and 1 mM UAAs, Nε-2-azidoethyloxycarbonyl-L-lysine (NAEK). To identify the UAA-dependence of PRV-PTC virus, a parallel packaging experiment was conducted in which the medium was not supplemented with UAA. The cells were further incubated at 37 °C in 5% CO_2_ until cytopathic effect (CPE) or syncytium was observed.

### 2.7. Electron Microscopy for PRV-PTC Virus

HEK-293T cells were transfected with plasmids described above for conventional electron microscopy analysis. Then when the cytopathic effect (CPE) or syncytium was observed, cells were fixed with 2.5% (*w*/*v*) glutaraldehyde in 200 mM HEPES (pH 7.4) for 2 h at room temperature, followed by post-fixation with 1% OsO_4_ and 1.5% K_3_Fe(CN)_6_ in H_2_O at 4 °C for 30 min. According to standard procedures, samples were dehydrated with acetone and impregnated with epoxy at room temperature and further embedded overnight at 70 °C for polymerization. Then the samples were cut into 70 nm ultrathin sections by ultrathin slicer (Leica, Wetzlar, Germany), stained with 2% uranium acetate for 17 min, lead citrate for 12 min. Specimens were examined using a conventional transmission electron microscope (TEM, h7650, Hitachi, Tokyo, Japan).

### 2.8. pB513B-Puro-MbpylRS-12 tRNA^Pyl^ Plasmid Construction

*MbpylRS* gene was optimized to make it more suitable for the mammalian cell system. The original plasmid pB513B was modified to obtain the pB513B plasmid containing MbpylRS and tRNA^Pyl^ simultaneously. Puromycin fragment was amplified by Puro (XbaI) -F: 5-ATTTTCTAGAATGACCGAGTACAAGCCACG-3, puro (NheI-EcoRI-HpaI) -R: 5-GCGTTAACGGTTGAATTCGTCGCTAGCGCGCTTGGGTC-3. *Puromycin* gene and the enzyme sites (NheI/EcoRI/HpaI) were inserted pB513B by enzyme digestion with XbaI/HpaI and named pB513B-Puro. The chicken β-actin promoter of the pCAGGS vector was amplified and digested by NheI/EcoRI to insert into pB513B-Puro. Subsequently, the optimized *MbpylRS* gene was constructed between EcoRI and HpaI to form a pB513B-Puro-MbpylRS plasmid. Furthermore, pB513B-Puro-MbpylRS plasmid was digested by SpeI/SfiI, and a pair of small gene sequences F: 5-GTCTTCCCAATCCTCCCCCTTGGATCCGACGTCAGCGTTCGTCGAC-3, R: 5-CTAGGTCGACGAACGCTGACGTCGGATCCAAGGGGGAGGATTGGGAAGACTGG-3 were annealed into small fragments and directly inserted into the digested vector pB513B-Puro-MbpylRS. The new vector was named pB513B-Puro-MbpylRS-mid, which contains the homologous arm of 12tRNA. Finally, pB513B-Puro-MbpylRS-mid was digested with BamHI, and 12tRNA fragments were obtained by recovering large fragments by gel electrophoresis after SalI/BbsI digestion of the bjmu-12t-zeo vector. Then, 12 tRNA^Pyl^ copies were constructed into pB513B-puro-MbpylRS-12-tRNA plasmid by homologous recombination. The newly constructed plasmid was named pB513B-puro-MbpylRS-12tRNA. Thus, a plasmid system was constructed simultaneously expressing puromycin, MbpylRS, and multiple tRNA^Pyl^ copies on a transposable plasmid.

### 2.9. Construction of GFP^39TAG^ Reporter Adenovirus

To generate adenovirus harboring GFP^39TAG^ reporter, HEK293 cells were transiently co-transfected with the pDC-315GFP^39TAG^ (plasmid with the TAG stop codon in *GFP* gene) with pBHGloxΔE1 and E3Cre helper plasmids as previously described [19]. 6 h after transfection, the medium was replaced with a fresh medium containing 2% FBS. The transfected cells were harvested after 7~9 d until plaque was observed.

### 2.10. Generation of Transgenic Cell Line Containing MbpylRS/tRNA^Pyl^ Orthogonal System

Indicated cells were seeded in 6 well plates and were co-transfected with 3 µg of pB513B-puro-MbpylRS-12tRNA plasmid and 1 µg of pB220A-1 plasmid using the transfection reagent jetPRIME (Polyplus, Illkirch, France). Non-transfected cells were used as control. Then, 6 h later, the transfection medium was replaced by DMEM medium supplemented with 10% FBS and 1 mM UAAs (NAEK). 48 h after transfection, the cells were selected under the pressure of indicated concentrate puromycin (Gibco, Waltham, MA, USA). The medium was replaced every day until the cells in the control group completely died. The resultant cells were stably transfected and continued to cultivate in the presence of 4 μg/mL puromycin. Then these cells were infected with GFP^39TAG^ reporter adenovirus in the presence of UAA, and the single clones were further sorted by fluorescence-activated cell sorting (FACS) according to the GFP reporter.

## 3. Results

### 3.1. Evaluation of UAA Site-Specific Incorporation for Potential PRV gB PTC Sites

**Figure 2:**
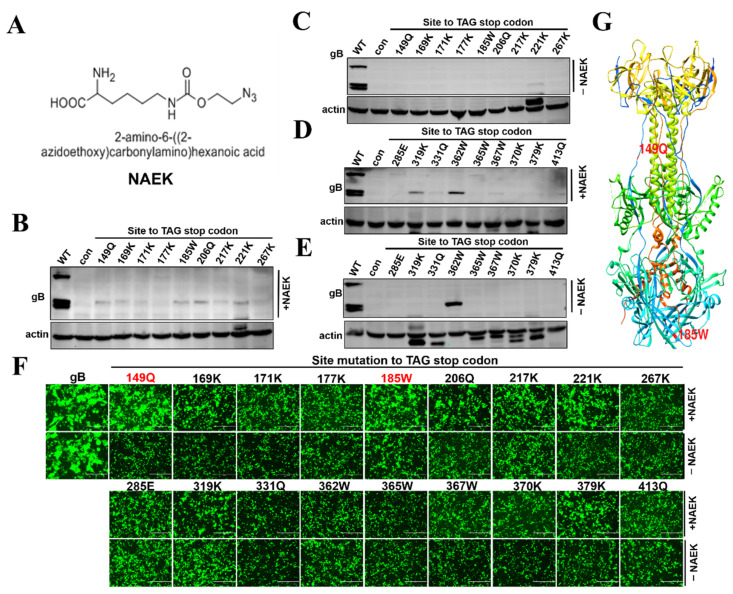
Evaluation of UAA site-specific incorporation for potential PRV gB PTC sites. (**A**) Chemical structure of Nε-2-azideoethyloxycarbonyl-L-lysine (NAEK). (**B**,**D**) HEK293T cells were co-transfected with GCE machinery and indicated gB-PTC plasmid; 6 h later, the fresh medium replaced the supernatant in the presence of 1 mM UAA (NAEK). Western blot was performed to analyze the UAA-dependent read-through efficacy of PTC harboring gB mutants in the presence of 1 mM UAA (NAEK) or (**C**,**E**) without UAA. β-actin was used as an internal control. (**F**) Cell-to-cell fusion assay was used to evaluate the read-through efficiency of PTC harboring gB mutants. RK13 cells were co-transfected with MbpylRS/tRNA^Pyl^, GFP, gD, gH, gL, gB, or its mutants, and 6 h later the supernatant was replaced by fresh medium supplemented with 2% FBS in the presence of 1 mM UAA (NAEK), or not. Fluorescence microscopy was used to analyze the read-through efficacy by indicating gB mediated cell fusion analysis at 48 h after transfection; the scale bar = 400 µm. The red labeled PTC constructs were used further study. (**G**) The corresponding position of 149 and 185 mutation points in the gB crystal structure.

*gB* was recognized as an essential gene for PRV replication. Therefore, we selected gB to engineer potential PTC sites. First, 149Q, 169K, 171K, 177K, 185W, 206Q, 217K, 221K, 267K, 285W, 319H, 331Q, 362W, 365W, 367W, 370K, 379K, 413Q of gB was separately engineered into amber codon (TAG). PTC containing gB constructs were confirmed by DNA sequencing (data not shown). Then the indicated gB-PTC constructs were co-transfected with the orthogonal MbpylRS/tRNA^Pyl^ pair plasmid, respectively. The UAA in this study was Nε-2-azidoethyloxycarbonyl-L-lysine (NAEK) as illustrated in Figure 2A. Full-length gB protein can be cleavaged by furin, as demonstrated in Figure 2B. Western blot result showed that gB was successfully expressed in the presence of 1 mM NAEK for indicated gB PTC constructs (Figure 2B,D). There was no gB expression without UAA (Figure 2C,E). This result indicated that some gB PTC sites read-through by GCE technology as expected, although the expression level was lower than wild-type gB (Figure 2B,D). As *gB*, *gD*, *gH*, and *gL* are the essential viral genes for virus-mediated cell-to-cell fusion, which is an important step for PRV spreading [20]. Next, a cell-to-cell fusion assay was used to test whether these NAEK incorporation sites influenced gB mediated cell-to-cell fusion. As previously described, a transient transfection-based cell-to-cell fusion assay was performed by co-transfection of PTC gB, gD, gH, gL and EGFP plasmids [18,21,22]. The result indicated that some gB PTC sites such as 149Q, 169K, 185W, 206Q, 379K successfully induced syncytia formation in the presence of NAEK, and no syncytia was formed without NAEK (Figure 2F). These results indicated that the effect of substitution by NAEK on function varies with position, and the position of 149 and 185 was labeled in the gB crystal structure (Figure 2G).

### 3.2. Construction and Rescue of the PTC Site Harboring PRV

**Figure 3:**
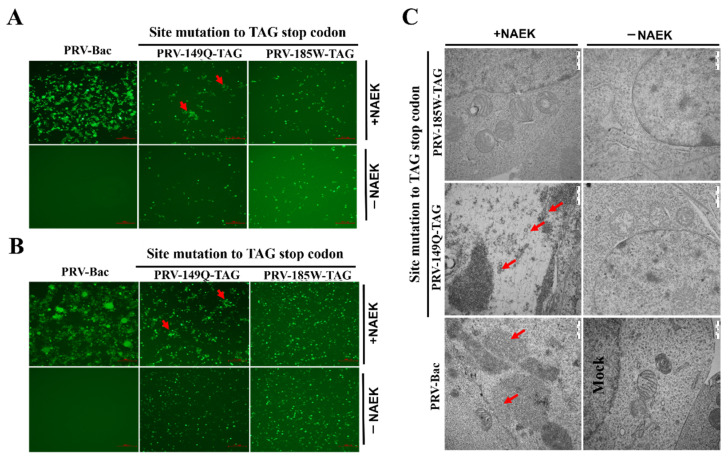
Rescue of PRV-PTC virus on HEK-293T and Vero cells. 2 µg MbpylRS /tRNA^Pyl^ plasmid was co-transfected with the 2 µg pPRV-Bac or the indicated pPRV-PTC-Bac (pPRV-149Q-TAG or pPRV-185W-TAG). At 6 h post-transfection, the supernatant was replaced with DMEM containing 2% FBS in the presence of 1 mM UAA (NAEK), or not. The rescue of PRV-PTC virus on (**A**) Vero cells or (**B**) HEK-293T cells was analyzed by fluorescence microscopy at 48 h post-transfection. The scale bar = 200 µm. (**C**) Electron microscopic analysis was performed to confirm the virus particles of the PRV-PTC virus. The scale bar = 1 µm.

Next, we selected 149Q and 185W as potential PTC engineering sites in PRV, pPRV-149Q-TAG and pPRV-185W-TAG were subsequently constructed using pBac-JS2012 and the galK selection system as previously described [15]. Vero cells were co-transfected with pPRV-149Q-TAG or pPRV-185W-TAG clones with plasmids containing orthogonal translation systems to rescue the PRV-PTC virus. At 48 h post-transfection, typical PRV-induced syncytia were formed in cells transfected with pPRV-149Q-TAG in the presence of UAA. No syncytia were formed without UAA (Figure 3A). However, pPRV-185W-TAG failed induced syncytia in the presence of UAA (Figure 3A). The same results were obtained in 293T cells (Figure 3B). To test whether infectious PRV particles were produced in the presence of UAA, electron microscopic analysis was performed (Figure 3C). Consistent with our above result, PRV particles were observed only in the pPRV-149Q-TAG transfected group in the presence of UAA. Furthermore, no PRV particles were observed in the control group and pPRV-185W-TAG transfected group (Figure 3C). Taken together, UAA could be used as a precise switch for controlling PRV-PTC virus replication.

### 3.3. Generation of MbpylRS/tRNA^Pyl^ Pair Delivery Vector and Reporter Adenovirus

**Figure 4:**
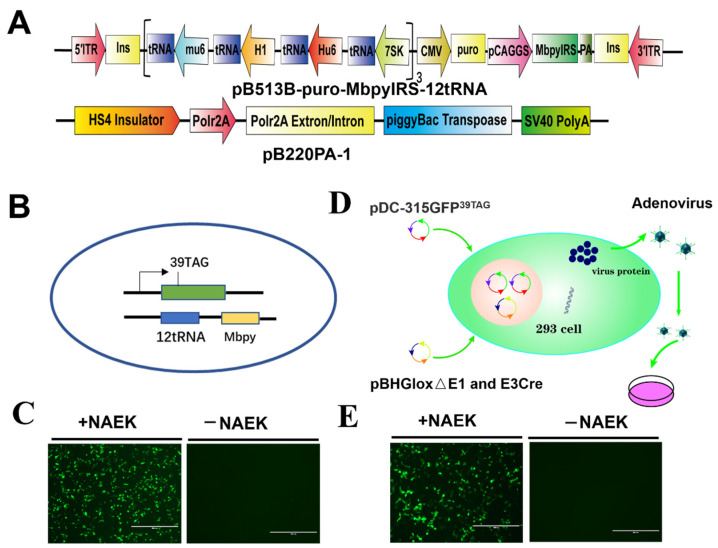
Generation of MbpylRS/tRNA^Pyl^ pair delivery vector and reporter adenovirus. (**A**) A PiggyBac transposon vector, pB513B-puro-MbpylRS-12tRNA, and a helper vector containing the PB transposase expression cassette, PB220A-1, were used to generate orthogonal transgenic stable cell lines. (**B**) *GFP* with an amber codon at position 39 was used as a reporter gene. (**C**) Functional GFP was visualized by fluorescence microscopy in the presence of the MbpylRS/tRNA^Pyl^ pair and UAA. Scale bars, 400 µm. (**D**) Schematic diagram of generating a recombinant adenovirus harboring *GFP^39TAG^* reporter gene. (**E**) Recombinant adenovirus was confirmed in RK13 cells; RK13 cells were first transfected with pB513B-puro-MbpylRS-12tRNA plasmid, then infected with recombinant adenovirus. The function of adenovirus was verified according to the expression of GFP^39TAG^. Scale bars, 400 µm.

An efficient method to incorporate UAA into the viral PTC site is to generate a stable transgenic cell line harboring MbpylRS/tRNA^Pyl^ pair in the host genome. Lentiviral vector and PiggyBac transposon system are powerful tools to generate stable cell lines [12,14]. However, the PiggyBac transposon system has the advantage of delivering large and complex DNA fragments into the genome of mammalian cells [23]. Therefore, in this study, we used the PiggyBac transposon system to deliver the MbpylRS/tRNA^Pyl^ cassette. First, a PiggyBac transposon vector, pB513B-puro-MbpylRS-12tRNA, was constructed. It contained MbpylRS, which was promoted by chicken β-actin promoter, and 12 tandem tRNA-expression cassettes promoted by U6 or H1 promoters (Figure 4A). To test whether this vector work normally, pB513B-puro-MbpylRS-12tRNA plasmid co-transfected with an amber codon-containing green fluorescent protein (GFP^39TAG^) reporter plasmid present, with or without UAA (Figure 4B). The results showed that functional GFP was visualized in the presence of UAA (Figure 4C), indicating pB513B-puro-MbpylRS-12tRNA was successfully constructed. To construct transgenic cells without reporter genes, we generate a recombinant adenovirus harboring GFP^39TAG^ (Figure 4D). Recombinant adenovirus was confirmed in RK13 cells, which were first transfected with pB513B-puro-MbpylRS-12tRNA plasmid, then infected with recombinant adenovirus. The results showed that functional GFP was visualized in cells supplemented with UAAs, indicating that recombinant adenovirus was successfully generated (Figure 4E).

### 3.4. Generation of Stable Cell Line Harboring GCE Machinery

**Figure 5:**
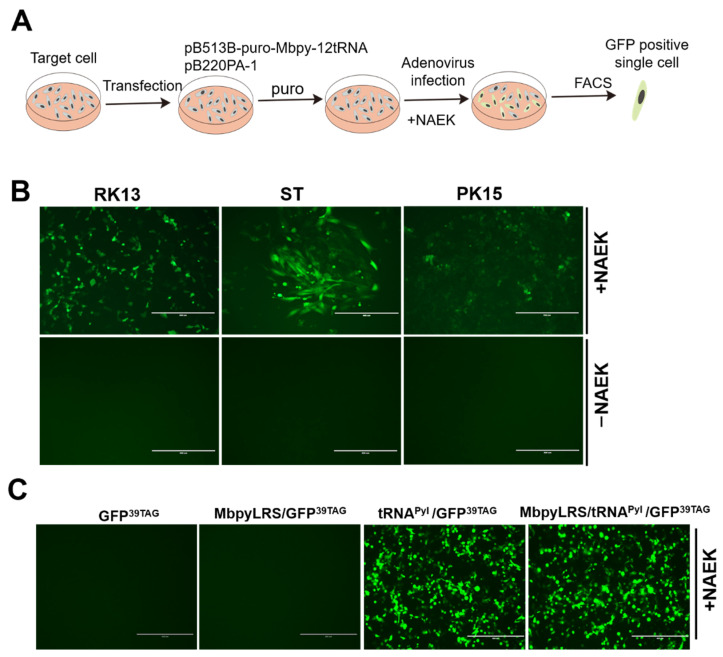
Generation of transgenic cell line containing MbpylRS/tRNA^Pyl^ orthogonal system. (**A**) Schematic representative of the process for generation of transgenic cells containing MbpylRS/tRNA^Pyl^ orthogonal system. (**B**) The well-growth transgenic RK13, ST, PK15 cell lines were infected with packaged adenovirus in the presence of UAA or not. The GFP^39TAG^ expression in these cells was observed by fluorescence microscopy. Scale bars, 400 µm. (**C**) Detection of the key limit factor for orthogonal system fail to translation in RK13 transgenic cell line by transfecting of MbpylRS, GFP^39TAG^ or tRNA^Pyl^ individually or together. The GFP^39TAG^ expression in these groups was observed by fluorescence microscopy. Scale bars, 400 µm.

To generate stable transgenic RK13, ST, and PK15 cell lines harboring GCE machinery, we co-transfected pB513B-puro-MbpylRS-12tRNA with pB220PA-1 (a vector expressing the PiggyBac transposase) together (Figure 4A). 48 h post-transfection, puromycin (4 μg/mL) was added. Two weeks later, puromycin-resistant cells were infected with reporter adenovirus in the presence of UAA, and GFP expressing single cells were further sorted by fluorescence-activated cell sorting technology (FACS) (Figure 5A). A single-cell of transgenic RK13, ST, PK15 cells was cultivated, to increase over approximately 2–3 weeks. Next, these cell lines were confirmed by infecting with GFP^39TAG^ adenovirus in the presence of UAA, or not. The GFP^39TAG^ expressed well in these cells in the presence of UAA (Figure 5B). The results demonstrated that MbpylRS/tRNA^Pyl^ pair could be successfully delivered by the PiggyBac transposon system.

To our surprise, unlike previous reports [12,14], all our transgenic cell lines are extremely unstable along with the increased passage. An overexpression assay was performed to test which element was lost in these cells. By transfecting MbpylRS, GFP^39TAG^ or tRNA^Pyl^ individually or together, and by co-transfection together, the group was used as a positive control (Figure 5C). Our result revealed that the tRNA^Pyl^ and GFP^39TAG^ co-transfection group restored robust and efficient GFP^39TAG^ expression in the presence of UAA. However, the GFP^39TAG^ transfection group and MbpylRS and GFP^39TAG^ co-transfection group failed to restore efficient GFP^39TAG^ expression in the presence of UAA (Figure 5C). Thus, we concluded that the expression of orthogonal tRNA is the key limiting factor in generating a stable cell line.

## 4. Discussion

In recent decades, GCE technology has been widely used to engineer PTC sites in essential genes to control different kinds of virus replication, such as HDV, HIV, Zika, FMDV [13,24,25,26,27]. Application of GCE technology in Influenza A virus makes it well-known as a novel tool for vaccine development [14]. In this study, we generated PTC harboring PRV, and the results suggested that PRV-PTC virus was successfully rescued in the presence of orthogonal system MbpylRS/tRNA^Pyl^ pair and UAA. This study was the first to demonstrate that UAA was incorporated into PRV gB protein by GCE technology. However, efficiency was generally low. Western blot showed different degrees of weak protein expression in 149Q, 169K, 185W, 206Q, 379K PTC sites, indicating that the incorporation of UAA may have a site preference (Figure 2B). However, only a few of these manifested the UAA-dependent gB mediated cell-to-cell fusion phenotype (Figure 2F). So we speculated that UAA incorporation in target proteins might have deleterious effects on gB function for some PTC sites [28]. More PRV essential genes and potential PTC sites should be screened in the future to obtain ideal PTC sites with efficient, site-specific incorporation of UAAs into PRV.

According to the Western blot and cell-to-cell fusion assay, we chose 149Q and 185W as potential sites to engineer amber codon TAG based on the pPRV-Bac infectious clone and successfully generated pPRV-149Q-TAG or pPRV-185W-TAG PTC virus. pPRV-149Q-TAG PTC virus was successfully rescued in the presence of UAA, demonstrating that GCE technology could control PRV PTC replication in vitro. Unfortunately, in our study the viral titer is extremely low, making it difficult to perform animal experiments in order to evaluate its efficacy as a potential vaccine. pPRV-185W-TAG PTC virus failed to rescue in the presence of UAA. We envisaged that the surrogate of tryptophan at position 185 by UAA might destroy other functions besides a cell-to-cell fusion of gB protein. Therefore, we concluded that the structure of UAA and the incorporation sites might influence the protein function.

Previous reports have demonstrated that the lentivirus vector [13,14] or PiggyBac transposon system successfully constructed stable cell lines harboring the orthogonal translation system [12,13]. Efficient incorporation of UAA requires multiple copies of tRNA^Pyl^ [29], which makes it difficult to package lentivirus efficiently [30]. The PiggyBac transposon system is characterized by rapid and efficient integration of large and complex sequences into mammalian cells’ genomes [23,31]. Therefore, this study used the PiggyBac transposon system to generate cell lines containing orthogonal translation machinery. Unfortunately, unlike the previous reports [12,13,14], all our attempts failed to generate a stable orthogonal MbpylRS/tRNA^Pyl^ system in the current work. The result indicated that insufficient orthogonal tRNA^Pyl^ copies were the limitation steps. Wolfgang H. Schmied et al. developed an optimized pyrrolysyl-tRNA synthetase/tRNA_CUA_ expression system and engineered eukaryotic release factor subunit 1 (eRF1) to efficient incorporate UAA in mammalian cells [29]. Future work will investigate the optimized approaches to efficiently incorporating UAAs at PTC sites in eukaryotic cells.

## 5. Conclusions

In conclusion, we demonstrated that an orthogonal translation system-mediated amber codon suppression strategy could precisely control PRV-PTC virus replication. To our knowledge, this is the first study reported for herpesvirus generated by GCE technology. However, there are still many challenges remaining to be addressed. Our further work will establish transgenic cell lines with high-efficiency expression of the orthogonal translation system.

## Data Availability

Data is contained within the article or supplementary material. The data presented in this study are available in the insert article or supplementary material here.

## Supplementary Materials

The following supporting information can be downloaded at: https://www.mdpi.com/article/10.3390/v14030572/s1, Table S1: primers for constructing PTC harboring gB mutants.
