# Supplementary material for: Generation of Premature Termination Codon (PTC)-Harboring Pseudorabies Virus (PRV) via Genetic Code Expansion Technology
Source: Viruses. 2022 Mar 10;14(3):572. doi: 10.3390/v14030572 (PMC8950157; doi:10.3390/v14030572)
Supplement: Supplementary file 1 [file viruses-14-00572-s001.zip › viruses-1622083-supplementary/Table S1.pdf]

TABLE S1. Primers for construction of PTC harboring gB mutants.

Primer	sequence (5'-3')
gB-149Q-TAG-F	TGCCCCGAGTACTCGTAGGGGCGCAACTTCACG
gB-149Q-TAG-R	CGTGAAGTTGCGCCCCTACGAGTACTCGGGGCA
gB-169K-TAG-F	AACATCGCCCCGCACTAGTTCAAGGCCCACATC
gB-169K-TAG-R	GATGTGGGCCTTGAACTAGTGCGGGGCGATGTT
gB-171K-TAG-F	GCCCCGCACAAGTTCTAGGCCCACATCTACTAC
gB-171K-TAG-R	GTAGTAGATGTGGGCCTAGAACTTGTGCGGGGC
gB-177K-TAG-F	GCCCACATCTACTACTAGAACGTCATCGTCACG
gB-177K-TAG-R	CGTGACGATGACGTTCTAGTAGTAGATGTGGGC
gB-185W-TAG-F	ATCGTCACGACCGTGTAGTCCGGGAGCACGTAC
gB-185W-TAG-R	GTACGTGCTCCCGGACTACACGGTCGTGACGAT
gB-206Q-TAG-F	GTGCCCGTCCCCGTGTAGGAGATCACGGACGTG
gB-206Q-TAG-R	CACGTCCGTGATCTCCTACACGGGGACGGGCAC
gB-217K-TAG-F	ATCGACCGCCGCGGCTAGTGCGTCTCCAAGGCC
gB-217K-TAG-R	GGCCTTGGAGACGCACTAGCCGCGGCGGTCGAT
gB-221K-TAG-F	GGCAAGTGCGTCTCCTAGGCCGAGTACGTGCGC
gB-221K-TAG-R	GCGCACGTACTCGGCCTAGGAGACGCACTTGCC
gB-267K-TAG-F	AACGACACCTACACCTAGATCGGCGCCGCGGGC
gB-267K-TAG-R	GCCCGCGGCGCCGATCTAGGTGTAGGTGTCGTT
gB-258W-TAG-F	CTCGGCACCCGCGGCTAGCACACCACCAACGAC
gB-258W-TAG-R	GTCGTTGGTGGTGTGCTAGCCGCGGGTGCCGAG
gB-319H-TAG-F	CTGCGCGAGGGGGCCTAGGGGGAGCACATCGGC
gB-319H-TAG-R	GCCGATGTGCTCCCCCTAGGCCCCCTCGCGCAG
gB-331Q-TAG-F	GCGCCCGGGCGCTTCTAGCAGGTGGAGCACTAC
gB-331Q-TAG-R	GTAGTGCTCCACCTGCTAGAAGCGCCCGGGCGC
gB-365W-TAG-F	CACTTCACGGTGGCCTAGGACTGGGCCCCCAAG
gB-365W-TAG-R	CTTGGGGGCCCAGTCCTAGGCCACCGTGAAGTG
gB-367W-TAG-F	ACGGTGGCCTGGGACTAGGCCCCCAAGACGCGG

gB-367W-TAG-R	CCGCGTCTTGGGGGCCTAGTCCCAGGCCACCGT
gB-370K-TAG-F	TGGGACTGGGCCCCCTAGACGCGGCGCGTGTGC
gB-370K-TAG-R	GCACACGCGCCGCGTCTAGGGGGCCCAGTCCCA
gB-379K-TAG-F	GTGTGCAGCCTGGCCTAGTGGCGCGAGGCCGAG
gB-379K-TAG-R	CTCGGCCTCGCGCCACTAGGCCAGGCTGCACAC
gB-413Q-TAG-F	GTCAGCGACGTCACGTAGCTCGACCTGCAGCGC
gB-413Q-TAG-R	GCGCTGCAGGTCGAGCTACGTGACGTCGCTGAC
